# To Our Patrons

**Published:** 1867-09

**Authors:** 


### TO OUR PATRONS.

The *Journal* has now reached the Seventh number of the Second Volume. Thus it is that nineteen numbers have been sent out to our readers. The terms require cash, in advance; but owing to the great pressure upon the country, pecuniarily and otherwise, since the war, many have been furnished with the Journal regularly at considerable labor and expense without advance payment. We have suffered financial embarrassment, more or less, during the whole time that we have thus labored assiduously to prepare matter, and pay for publication. We do not make this appeal for advance payments particularly, but that we may have remittance made of *debts* due us for what has already been received and used. We say to our readers, will you allow us to be ruined, when you can avert it by mailing to the Editors at their risk, the small pittance of *four dollars ! ?* Honor, humanity, and every thing sacred in your heart say, no! The time has been, and recently, too, when those, unaccustomed to want, hitherto, could rarely meet with money at all ; but now, since the country has been blessed with a bountiful harvest of wheat, and the prospect of a fine yield of corn and cotton, the readers of Medical Journals, will be able to obtain from their patrons remuneration for services, and relieve their distressed creditors, at least to the small extent our demands go. We take courage at the very thought. Do remit, if possible, during the month of September.

